# Tumor Budding in Upper Gastrointestinal Carcinomas

**DOI:** 10.3389/fonc.2014.00216

**Published:** 2014-08-14

**Authors:** Viktor H. Koelzer, Rupert Langer, Inti Zlobec, Alessandro Lugli

**Affiliations:** ^1^Clinical Pathology Division, Institute of Pathology, University of Bern, Bern, Switzerland; ^2^Translational Research Unit, Institute of Pathology, University of Bern, Bern, Switzerland

**Keywords:** gastrointestinal cancer, gastric cancer, esophageal cancer, tumor microenvironment, tumor budding, prognostic factor, epithelial–mesenchymal transition

## Abstract

The basis of personalized medicine in oncology is the prediction of an individual’s risk of relapse and death from disease. The presence of tumor budding (TB) at the tumor–host interface of gastrointestinal cancers has been recognized as a hallmark of unfavorable disease biology. TB is defined as the presence of dedifferentiated cells or small clusters of up to five cells at the tumor invasive front and can be observed in aggressive carcinomas of the esophagus, stomach, pancreas, ampulla, colon, and rectum. Presence of TB reproducibly correlates with advanced tumor stage, frequent lymphovascular invasion, nodal, and distant metastasis. The UICC has officially recognized TB as additional independent prognostic factor in cancers of the colon and rectum. Recent studies have also characterized TB as a promising prognostic indicator for clinical management of esophageal squamous cell carcinoma, adenocarcinoma of the gastro-esophageal junction, and gastric adenocarcinoma. However, several important issues have to be addressed for application in daily diagnostic practice: (1) validation of prognostic scoring systems for TB in large, multi-center studies, (2) consensus on the optimal assessment method, and (3) inter-observer reproducibility. This review provides a comprehensive analysis of TB in cancers of the upper gastrointestinal tract including critical appraisal of perspectives for further study.

## Introduction

The foundation of personalized oncological therapy is the prediction of an individual’s risk of relapse and death from disease. Staging of tumors is performed according to the AJCC/UICC TNM classification (1). Following these internationally accepted guidelines, therapy decisions are made based on the anatomic extent of the tumor as determined by clinical, radiographic, and pathologic staging. In carcinomas of the upper gastrointestinal tract, we know that most patients with early invasive disease can expect a good prognosis and consequently advocate surgical or endoscopic resection as standard of care, while patients with locally advanced tumors or nodal metastasis are treated with multimodality therapy (2). Treatment decisions carry severe consequences for the patient. Precise risk assessment is therefore of central importance to balance benefit and overtreatment.

**Figure 1:**
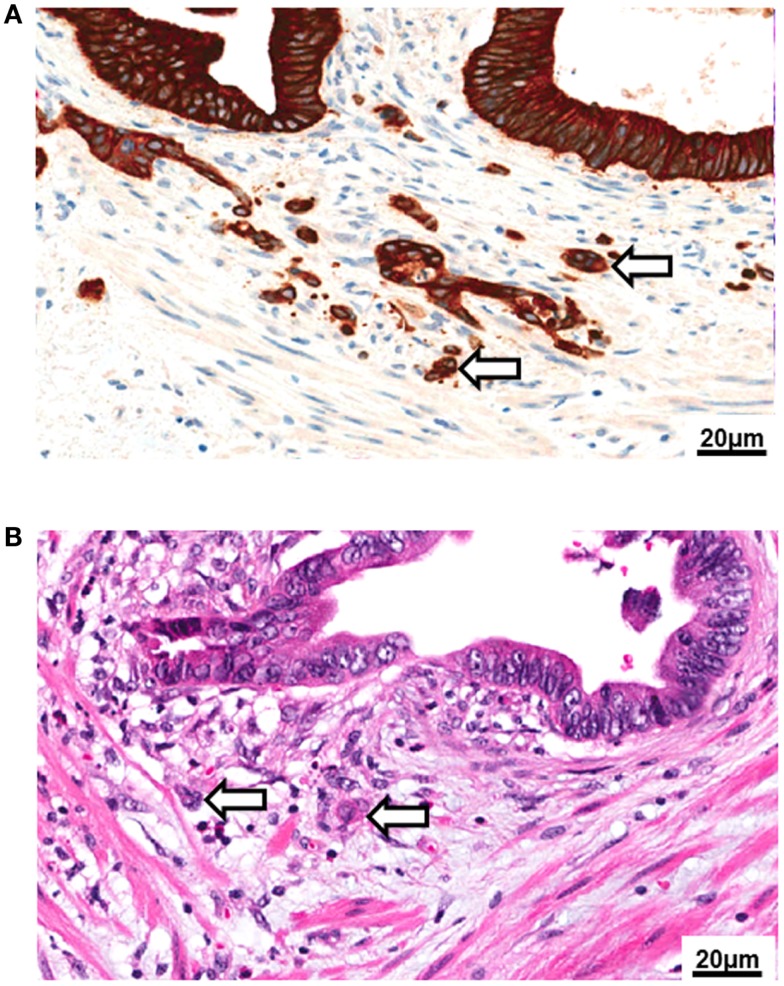
**Tumor budding**. **(A)** High-power image (250×) of tumor budding at the invasive front of colorectal cancer as visualized by pancytokeratin (brown) immunohistochemistry. **(B)** Tumor buds as seen in a standard hematoxylin and eosin (H&E) stain (250×).

Following the TNM classification, patients with carcinomas of the upper gastrointestinal tract can be assigned to one of five main stages (1, 3). However, in the light of precision medicine and molecular pathology, risk stratification based only on the T, N, M, L, V, and R classifiers has to be viewed as approximate. In cancers of the upper gastrointestinal tract, we know that unexpected adverse clinical outcomes can be observed in patients even with early-stage disease (4, 5). Additional prognostic indicators based on tumor and host characteristics are therefore needed to find a precision approach. In particular, tumor-related factors found in the microenvironment may centrally impact patient prognosis. Recent studies highlight that epithelial tumors undergo mesenchymal transition (EMT) in the tumor microenvironment, allowing dissociative growth, migration, and lysis of stromal components (6–8). In carcinomas of the gastrointestinal tract, the visible correlate of cells undergoing EMT is thought to be the presence of single cells or small clusters of up to five cells in the tumor stroma ahead of the invasive front, termed tumor budding (TB) (6, 7, 9, 10) (Figure 1). It is important to note that tumor buds can be quantified on the histologic slide to provide a TB score, which correlates with tumor aggressiveness. The prognostic value of TB has been established in a large number of independent retrospective studies for squamous cell carcinoma (SCC) (11–15) and adenocarcinoma of the esophagus (16–19), stomach (20, 21), colon (22–25), and rectum (26–29). Importantly, TB is officially recognized as an additional prognostic factor by the UICC for colorectal cancer and is listed by the European (30) and Japanese (31) guidelines for colorectal cancer screening and diagnosis as well as the guidelines of the European Society for Medical Oncology (ESMO) (32). Further, TB has recently been listed as a non-core data item by the European consensus conference colon and rectum (EURECCA), highlighting the increased use of this feature in clinical practice (33).

**Figure 2:**
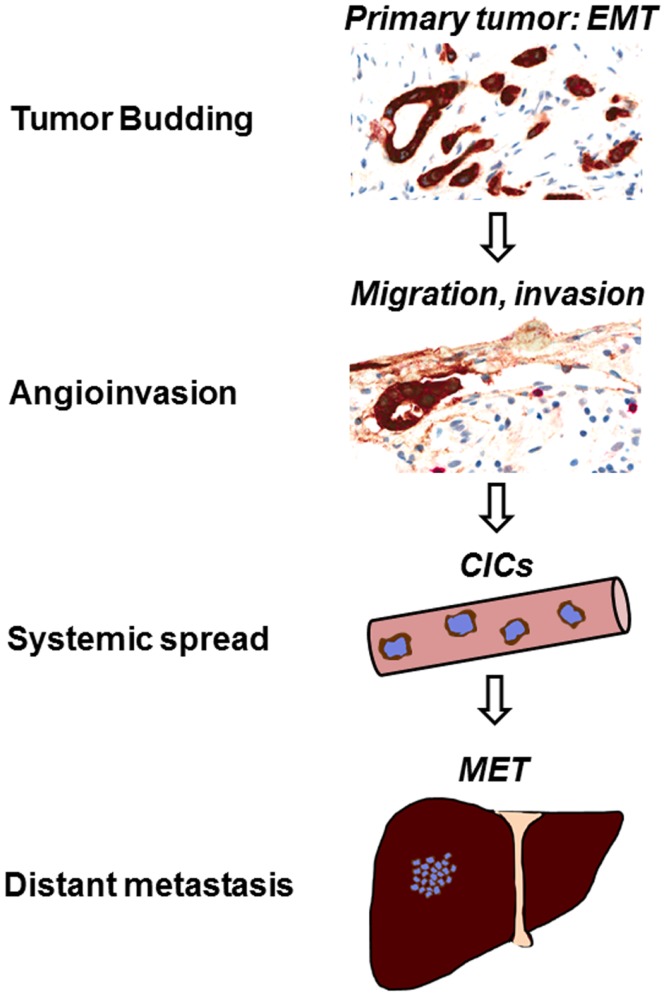
**Tumor budding leads to vascular invasion and metastasis**. The formation of tumor buds is thought to be the visible correlate of epithelial–mesenchymal transition (EMT) in the tumor microenvironment. Following tumor cell dissociation, lymphovascular invasion is frequently observed in high-grade budding tumors. TB could be a source of circulating cancer cells (CICs), leading to the establishment of metastasis at distant sites in the process of mesenchymal–epithelial transition (MET).

Biologically, tumor buds may transiently acquire a mesenchymal phenotype due to loss of E-cadherin expression and nuclear translocation of β-catenin leading to activation of WNT signaling (6, 34). Upregulation of metalloproteinases, urokinase receptor, and cathepsin may contribute to an increased migratory capacity and stromal invasion (35, 36). It has been suggested that tumor buds may resist both apoptosis and anoikis following the detachment from the primary tumor due to upregulation of anti-apoptotic proteins such as RAF-kinase inhibitor protein (RKIP) and caspase-3 deficiency (37–39). Further, tumor buds exhibit a low proliferative activity, which is in line with their migratory phenotype (38, 40). Taken together, these features may explain an increased resistance of tumor buds to chemo- and radiotherapy. Indeed, high-grade TB has been shown to be a highly specific indicator of poor response to radiotherapy in rectal cancer patients (41). As immunogenic cell death also entails the induction of apoptosis, this may also indicate an increased resistance to immunotherapeutic agents. Few studies have investigated the immunogenicity of tumor buds, but it has been suggested that loss of MHC-I expression could be a frequent feature in cancer initiating cells (42). CD8+ T-cells are frequent in the tumor microenvironment (43), yet may not be able to efficiently recognize tumor buds if MHC-I is lost. Interestingly, tumor buds may also possess stem cell like features, as suggested by an increased expression of ABCG-5 protein (44). These stem cell characteristics may allow cancer initiation at distant sites (Figure 2).

The formation of tumor buds by solid tumors may therefore represent an important first step toward metastasis. Interestingly, the histopathologist can visualize this step and make this feature accessible as a prognostic indicator for clinical management. In comparison to tumor grade, which has shown poor inter-observer reproducibility, the quantitative assessment of TB holds a definite advantage, just as mitoses are counted within a defined visual field for the histopathological reporting of the BRE-score score in breast cancer, tumor buds can be directly quantified under the microscope and reported as a quantitative feature. However, a broad spectrum of methods has been recommended for the assessment of TB in daily diagnostic practice. Morodomi (45) and Hase (46) have proposed a qualitative assessment based on the observers’ impression of TB as present or absent (Morodomi), none/mild or moderate/severe (Hase). Nakamura and colleagues recommend semi-quantitative assessment of the proportion of the invasive front with TB (23, 47). This highly subjective methodology is contrasted by quantitative scoring methods as illustrated by Ueno (22, 48), Wang (24), Ono (49), Park (50), Lugli (51), Ohike (52), and Karamitopoulou (25). A central advantage to counting tumor buds is that quantitative scores tend to have a much higher inter-observer reproducibility (53) (Table 1).

**Table 1:** **Methods used for assessment of tumor budding**.

Reference	Method	Classification	Staining	Region	Area
Morodomi et al., 1989 (45)	Qualitative; subjective assessment of TB intensity	Present or absent	H&E	Invasive front	Entire invasive front
Hase et al., 1993 (46)	Qualitative; subjective assessment of TB intensity	None/mild (BD-1)	H&E	Invasive front	Entire invasive front
		Moderate/severe (BD-2)	
Ono et al., 1996 (49)	Quantitative; all cancer cells with a single or solitary trabecular form with indistinct polarity (“focal dedifferentiation units”) counted along the invasive front (200×)	None (0 unit) Mild (1–20 units) Moderate (21–50 units) Severe (>50 units)	H&E	Invasive front	Entire invasive front
Nakamura et al., 2005 (47)	Semi-quantitative assessment of the proportion of the invasive front with TB	None, mild (<1/3 with TB) Marked (>2/3 with TB)	H&E	Invasive front	Entire invasive front
		Moderate (1/3–2/3 with TB)	
Park et al., 2005 (50)	Quantitative assessment of TB; the number of buds is counted in three fields assessed under high-power (200×) in area of most intense TB along invasive front. TB intensity is defined as maximum number of buds within the three fields	Continuous score	H&E	Invasive front	Entire invasive front assessed under low power, 1 field (200×) counted
Nakamura et al., 2008 (23)	Semi-quantitative assessment of the proportion of the invasive front with TB	None/mild (low-grade) Moderate/marked (high-grade)	H&E	Invasive front	Entire invasive front
Ueno et al., 2002 (48)	Quantitative; invasive front scanned at low power to identify region with densest TB; buds are counted in one HPF	Low-grade (<10 buds) High-grade (≥10 buds)	H&E	Invasive front	0.385 mm^2^
Ueno et al., 2004 (22)	Quantitative; invasive front scanned at low power to identify region with densest TB; buds are counted in one HPF	Low-grade (<5 buds) High-grade (≥5 buds)	H&E	Invasive front	0.785 mm^2^
Wang et al., 2009 (24)	Conventional method: quantitative; invasive front scanned at low power to identify region with densest TB; buds are then counted in 5 high-power fields	Low-grade (<50% of HPFs exceed the median bud count of all fields) High-grade (≥50% of HPFs exceed the median bud count of all fields)	H&E	Invasive front	0.94985 mm^2^
Wang et al., 2009 (24)	Rapid method: quantitative; 5 HPFs are evaluated for presence of TB	Low-grade (<50% of HPFs examined positive for TB)	H&E	Invasive front	0.94985 mm^2^
		High-grade (≥50% of HPFs examined positive for TB)	
Lugli et al., 2009 (1HPF method) (51)	Quantitative; 1 HPF counted in areas of densest TB	Low-grade (<10 buds in 1 HPF of highest density)	PanCK	Invasive front	0.49 mm^2^
		High-grade (≥10 buds in 1 HPF of highest density)	
Ohike et al., 2010 (52)	Semi-quantitative; budding foci are identified. Buds are counted in one HPF of each focus as specified by Ueno (48). A HPF is counted as positive for TB when 5 or more tumor buds are present	Low budding (0–2 positive fields) High budding (three or more budding fields)	H&E	Invasive front	0.785 mm^2^/HPF
Karamitopoulou et al., 2013 (10HPF method) (25)	Quantitative; 10 HPF counted in areas of densest TB	Low-grade (<100 buds total in 10 HPFs of highest density) High-grade (≥100 buds total in 10 HPFs of highest density)	PanCK	Invasive front	0.49 mm^2^/HPF for a total area of 4.9 mm^2^
Landau et al., 2014 (19)	Semi-quantitative; 5 HPFs are evaluated for presence of TB; A HPF is counted as positive for TB when 5 or more tumor buds are present	No budding (no budding fields) Focal budding (one to two budding fields)	H&E	Invasive front	0.785 mm^2^/HPF
		Extensive budding (three or more budding fields)			

*TB, tumor budding; HPF, high-power field; H&E, hematoxylin and eosin; PanCK, pancytokeratin*.

Taken together, high-grade TB in gastrointestinal carcinomas could be a general indicator of aggressive clinicopathological behavior with importance for individualized risk assessment. While many studies have addressed TB in colon and rectal carcinoma, a uniting perspective on TB in carcinomas of the upper gastrointestinal tract has so far not been attempted. Subject of this review is therefore to provide a comprehensive overview on TB as a novel and promising tumor-related histomorphological prognostic factor in cancers of the esophagus and stomach.

### Methods

The focus of this work was to review the primary and secondary literature on TB in upper gastrointestinal carcinomas. Electronic keyword searches using Boolean operators were performed to identify relevant primary sources in the following databases: MEDLINE, MEDLINE In Process, Scopus, Web of Science, EMBASE, Google Scholar, and ISI Proceedings. Manual searches were performed of the reference lists. Searches were not limited by date and include all literature published up to the 1st of July, 2014. Case reports or notes were excluded. All identified studies were reviewed and assessed for relevance. Data from relevant publications were extracted and categorized according to tumor type and location. Data extraction tables were reviewed by all four authors of this manuscript.

## Tumor Budding in Carcinomas of the Esophagus and Stomach

### Esophageal carcinoma

Carcinomas of the esophagus are the sixth most common malignancy worldwide in males and the fifth most common cause of death (15). Esophageal cancer occurs in two major histological types: SCC most frequently arises in the upper esophagus and accounts for up to 90% of esophageal carcinomas in developing countries (1). Esophageal adenocarcinoma, frequently originates from pre-malignant Barrett Mucosa (“Barrett’s carcinoma”), occurs in the lower third of the esophagus and has rapidly increased in incidence in developed countries over the last decades (1). Advances in early detection and multimodality therapy have increased survival rates of patients with esophageal cancers making histopathological risk stratification an important tool for therapeutic decision making. This applies both to early cancers of the esophagus, which can be treated by endoscopic mucosal resection (EMR), radiofrequency ablation, or limited resection (Merendino’s procedure) as well as advanced disease requiring neo-adjuvant chemotherapy and esophagectomy.

#### Tumor budding as histomorphological prognostic factor in squamous cell carcinoma of the esophagus

In SCC of the esophagus, TB has been evaluated for risk stratification in different clinical settings (Table 2). In an analysis of 56 patients with surgically resected esophageal SCC of stages I–III, high-grade TB was identified by Roh and colleagues in 60.7% of cases (11). Increased TB predicted more advanced pT-stage, lymphovascular invasion (LVI), perineural invasion, larger tumor size, and circumferential resection margin involvement. Further, patients with high-grade TB had a reduced 3-year survival outcome of 30.7% after esophagectomy in comparison to 72.3% in patients with low-grade budding. These findings are supported by two subsequent publications [Ref. (12), *n* = 136; (14), *n* = 82], which describe high-grade TB in 56.1–60.2% of patients undergoing esophagectomy for stage I–IV esophageal SCC (12, 14). Even though both authors included a significant subset of patients receiving pre-operative and/or adjuvant chemotherapy, both studies independently confirm TB as an indicator of poor survival outcome with 5-year survival rates of 35.0–35.4% in patients with high-grade TB as compared to 81.3–86.9% in absence of this feature. Further, high-grade TB consistently predicted more advanced T-stage and lymph node metastasis (12, 14). Miyata and associates addressed TB in the neo-adjuvant setting using a cohort of 74 stage II–IV patients. Interestingly, TB was also found to be a useful prognostic indicator in patients who received neo-adjuvant chemotherapy followed by surgery for advanced esophageal SCC (13). In particular, the 5-year survival rate of patients with high-grade TB in the resection specimen was 17%, compared with 49% for those with low-grade TB. Further, TB correlated significantly with a poor clinical chemotherapy response.

**Table 2:** **Studies on tumor budding as a histomorphological prognostic factor in squamous cell carcinoma of the esophagus**.

Reference	Tumor subtype	Stage	*N*	Treatment	Method for assessment of TuB	Low-grade/high-grade TB%	Correlation of high-grade TB with clinicopathological features (*p* < 0.05)	Outcome
Roh et al., 2004 (11)	SCC	I–III	56	56/56 Patients were treated by primary resection	Ueno et al., 2004 (22) H&E	39.3/60.7	T-stage, L1, Pn1, larger tumor size, circumferential resection margin involvement, advanced AJCC-stage	3-year survival rates after esophagectomy with low-grade TB: 72.3%; high-grade TB: 30.7% (*p* = 0.04)
Koike et al., 2008 (12)	SCC	I–IV	136	82/136 Patients received pre-operative CTX- and/or RTX 52/139 Patients received adjuvant CTX- and/or RTX	Ueno et al., 2004 (22) H&E	39.8/60.2	T-stage, N-stage, L1, increased invasion depth, larger tumor size, intramural metastasis	5-year survival rates after esophagectomy with low-grade TB: 35.4%; high-grade TB: 81.3% (*p* = 0.001)
Miyata et al., 2009 (13)	SCC	II–IV	74	74/74 Patients received pre-operative CTX	Ueno et al., 2004 (22) H&E	59/41	Not analyzed	5-year survival rates after esophagectomy with low-grade TB: 49%; high-grade TB: 17% (*p* < 0.001)
Nakanishi et al., 2011 (14)	SCC	I–IV	82	82/82 Patients were treated by primary resection 22/82 Patients received adjuvant CTX	Ueno et al., 2004 (22) PanCK	43.9/56.1	T-stage, N-stage, L1, V1, larger tumor size	Disease-free survival time in patients with low-grade TB: 113 months; in patients with high-grade TB: 31 months (*p* < 0.0001)
Teramoto et al., 2013 (15)	SCC	I	79	73/79 Patients were treated by primary resection 6/79 Patients were treated by EMR followed by radical surgery	Modified Ueno et al., 2004 (22) H&E	63.3/36.7	N-stage, L1, V1, tumor differentiation, increased invasion depth	3-year survival rates after esophagectomy with low-grade TB: 94.5%; High-grade TB: 48.8% (*p* < 0.001)
				11/79 Patients received adjuvant CTX- and/or RTX				

*TB, tumor budding; SCC, squamous cell carcinoma; AC, adenocarcinoma; CTX, chemotherapy; RTX, radiotherapy; H&E, hematoxylin and eosin; PanCK, pancytokeratin; V1, venous invasion; L1, lymphatic invasion; Pn1, perineural invasion*.

Moreover, TB was confirmed as a decisive prognostic indicator in surgically treated early-stage esophageal SCC in a recent study including 79 pT1 patients by Teramoto and colleagues (15). In these early-stage patients, only 36.7% showed high-grade TB while 63.3% were assigned to the low-grade budding group; this data suggest that TB may be less frequently encountered in patients with limited disease. Following esophagectomy, the investigators report 3-year survival rates of 48.8% for pT1 patients with high-grade TB as compared to 94.5% in the absence of this feature. Within the cohort, 11 patients received adjuvant therapy.

In summary, these studies support the use of TB as a useful histomorphological prognostic factor for primarily resected esophageal SCC, in the neo-adjuvant setting and in early-stage cancers of the esophagus. The validation of TB as a histomorphological prognostic factor in squamous cell esophageal cancer in large multi-centric studies is therefore recommended.

#### Tumor budding as histomorphological prognostic factor in adenocarcinoma of the esophagus

Four studies were identified following a literature search examining TB in AC of the esophagus or gastro-esophageal junction (Barrett’s carcinoma) (Table 3). In a well-designed, two-center retrospective study including 287 cases of stage I–IV esophageal AC and 69 cases of SCC by Brown and associates, high-grade TB was associated with a median overall survival outcome of 15 months while patients with low-grade TB reached 31 months (16). Importantly, TB retained prognostic significance in both the AC and SCC subgroup (*p* = 0.0001 and *p* = 0.021, respectively), when including neo-adjuvant therapy (*n* = 115/356 patients; *p* = 0.003) and in multivariable analysis with age, *N*-stage, and overall-stage (*p* = 0.002). Further, high-grade TB was associated with higher overall TNM-stage and adverse clinicopathological features such as lymph node metastasis, poor tumor differentiation, and incomplete excision. However, no separate analysis of SCC and AC was attempted concerning TNM-relevant clinicopathological features. Of note, a recent study presented at the Annual Meeting of the German Society of Pathology confirmed the association of TB with aggressive histopathology of esophageal and GI-junction AC on an independent set of 86 stage I–IV patients treated by primary resection (17). In particular, high-grade TB correlated significantly with advanced T-stage, higher tumor grade, non-intestinal/diffuse histological subtype, and higher rates of R1-resection. Survival analysis showed a trend toward poor overall survival in patients with high-grade TB but did not reach statistical significance, possibly due to the relatively small number of patients under study.

**Table 3:** **Studies on tumor budding as a histomorphological prognostic factor in adenocarcinoma of the esophagus**.

Reference	Tumor subtype	Stage	*N*	Treatment	Method for assessment of TuB	Low-grade/high-grade TB%	Correlation of high-grade TB with clinicopathological features (*p* < 0.05)	Outcome
Brown et al., 2010 (16)	AC/SCC	I–IV	Total: 356 69 SCC 287 AC	241/356 Patients treated by primary resection 115/356 Patients received pre-operative CTX	Ueno et al., 2004 (22) H&E	48.3/51.7	T-stage, N-stage, poor tumor differentiation, circumferential resection margin involvement, higher overall TNM-stage, low inflammatory response. No separate analysis of SCC and AC was performed.	Low-grade TB: OS 31 months; high-grade TB: OS 15 months (*p* < 0.0001) Prognostic impact of TB is independent of histologic type (SCC, *p* = 0.021; AC, *p* = 0.0001), age, N-stage, overall stage
Thies et al., 2013 (17)	AC	I–IV	86	86/86 Patients were treated by primary resection	Karamitopoulou et al., 2013 (25) PanCK	60.5/39.5	pT-stage, higher tumor grade, non-intestinal/diffuse histological subtype, higher rates of R1-resection	Survival analysis showed a trend to worse survival for high-grade TB (*p* = 0.15; ITB *p* = 0.13)
Nowak et al., 2013 (18)	AC	I	42	42/42 Patients were treated by primary resection	Adapted Ueno et al., 2004 (22) H&E	Not specified	N-stage	High-grade TB was a strong predictor of tumor recurrence (HR = 14.21; *p* = 0.022) and reduced OS (HR = 3.06; *p* = 0.015)
Landau et al., 2014 (19)	AC	I esophageal AC or AC of gastro esophageal junction	210	210/210 Patients were treated by primary resection	Ueno et al., 2004 (22) H&E adapted according to Ohike et al., 2010 (52)	Any TB: 44.3% focal: 16% extensive 28% no TB: 55.7%	N-stage, submucosal invasion depth, grade, angioinvasion, tumor size	5-year OS: no TB: 79%, focal TB: 71%, extensive TB: 37% (*p* > 0.001) Multivariate analysis: HR for death (extensive TB): 3.3 (95% CI = 1.5–7.4, *p* = 0.004); independent of T- and N-stage, age, type of surgery
								Disease recurrence at 24 months: no TB: 5%, focal TB: 19%, extensive TB: 36%
								Multivariate analysis: HR for recurrence (extensive TB): 3.3 (95% CI = 1.4–7.0, *p* = 0.005); independent of T- and N-stage

*TB, tumor budding; SCC, squamous cell carcinoma; AC, adenocarcinoma; CTX, chemotherapy; RTX, radiotherapy; H&E, hematoxylin and eosin; PanCK, pancytokeratin; V1, venous invasion; L1, lymphatic invasion; Pn1, perineural invasion; OS, overall survival*.

Two independent studies specifically address the prognostic impact of TB in early-stage AC of the esophagus. In 42 patients with surgically treated pT1 AC, Nowak and colleagues demonstrate a significantly increased rate of tumor recurrence and nodal metastasis with high-grade TB (18). Further, high-grade TB predicted a high risk for adverse survival outcome (HR = 3.06; *p* = 0.015). These results were recently expanded in a well-designed retrospective analysis by Landau et al. for 210 surgically resected pT1 AC of the esophagus (19). The authors identify a significant correlation between TB-grade and submucosal invasion. In fact, 95% of tumors with extensive TB invaded the superficial (pT1a) or deep submucosal layer (pT1b) as compared to 54% of cases with none or focal TB (*p* < 0.001). Further, extensive TB correlated strongly with aggressive histopathological features, including poor tumor differentiation, angioinvasion, and larger tumor size (all *p* < 0.001). Even though the overall outcome is favorable in pT1 patients, Landau and colleagues report a survival rate of only 37% of patients with extensive TB at 5 years follow-up as compared to 79% in absence of this feature (*p* < 0.0001). In multivariable analysis, the negative prognostic impact of TB (HR 3.3; 95% CI: 1.5–7.4; *p* = 0.004) was found to be independent of submucosal invasion depth, N-stage, patient age, and type of surgery. Similar results were observed for tumor recurrence at 24 months after surgery; while only 5% of patients without TB relapsed, tumor recurrence was found in 19% of those with focal TB and 36% of patients with extensive TB (*p* < 0.0001). This was independent of both T- and N-stage in multivariable analysis (HR = 3.2; 95% CI = 1.4–7.0).

Taken together, these studies indicate that TB may be a valuable predictor of aggressiveness of disease in esophageal AC. Early esophageal carcinoma with lamina propria (pT1a) or submucosal invasion (pT1b) not infrequently take a detrimental course as a consequence of micrometastasis already present at the time of resection (54). EMR is an increasingly popular treatment option for the early-stage subgroup. Precise risk assessment in the surgical pathology practice is therefore of crucial importance for clinical management of early-stage AC. In locally advanced AC of the esophagus, TB in the resection specimen may serve as a valuable prognostic indicator to guide clinical follow-up. Large, multi-centric studies including robust statistical analysis are therefore recommended to validate the prognostic impact of TB in the clinical management of esophageal AC.

#### Future outlook

Opportunities for further study of TB seem abundant in esophageal cancer, as several important factors are unique to the assessment of histomorphological biomarkers – and particularly TB – in this disease. First, SCC is common in the esophagus but very rare in other locations of the GI-tract. As TB was initially defined as a histomorphological prognostic factor in the microenvironment of AC of the colon and rectum, the need of a systematic comparison of the biological characteristics of tumor buds in SCC of the esophagus and AC of the GI-tract is evident. A conserved pattern of features related to the process of EMT would further support the notion of tumor buds as histomorphological hallmark of aggressive disease biology in the tumor microenvironment.

Second, multimodality treatment strategies for esophageal cancers differ from cancers of the stomach and colorectum. While presence of TB in the surgically resected specimen of CRC may be an indicator of aggressive disease requiring adjuvant chemotherapy, esophageal cancers are commonly treated in the neo-adjuvant setting where no information on budding activity in the tumor microenvironment based on a resection specimen will be available. Consequently, novel predictive factors based on biopsy material of esophageal cancers are needed that could aid risk stratification in conjunction with imaging and clinical findings. Interestingly, intra-tumoral budding (ITB) may represent a novel prognostic feature present in pre-operative biopsies of cancers of the GI-tract, particularly of the colorectum (41, 55, 56). Based on the consistent association of TB with aggressive histomorphological features in both AC and SCC, investigation of ITB in esophageal carcinomas may be expected to provide similar information.

Third, no agreement on the optimal approach to assess TB in esophageal cancer has been found. For the visualization of TB, two studies using cytokeratin staining were identified (14, 17), while the remaining studies are based on standard H&E staining (11–13, 15, 16, 18, 19). Methodologically, TB in esophageal SCC has been assessed in one densest high-power field (HPF, 200×) according to Ueno (22) by eight authors with variable modifications. Other authors have used the scoring approach suggested by Ohike (52) and the 10 HPF methods (25) (Tables 2 and 3). Only one study was identified assessing inter-observer variability for TB using a 10 HPF approach (17). Consequently, a systematic inter-observer study comparing different methods for the assessment of TB seems desirable both in SCC and AC to reach consensus on the optimal approach for daily diagnostic practice.

### Gastric adenocarcinoma

Gastric cancer is among the five most frequent cancers in males and females worldwide with an estimated 738,000 deaths in 2011 (15). Gastric AC occurs in two major histological subtypes according to the Lauren classification, intestinal, and diffuse (1). As standard of care, operable patients with stage II–III disease are treated with multimodality therapy following resection (57–59). For stage IB patients with node positive (T1 N1) and muscle invasive disease (T2 N0) the role of adjuvant chemotherapy is less clear. Even though the prognosis of early-stage patients is relatively favorable following complete resection of the primary tumor, individual patients may follow an aggressive clinical course characterized by local and distant disease recurrence (60). Additional prognostic factors such as TB may be particularly helpful for risk stratification in this setting to identify patients that may profit from multimodal treatment following resection.

#### Tumor budding as an adverse histomorphological prognostic factor in gastric adenocarcinoma

Literature analysis of TB in gastric AC identified only two studies from independent centers (Table 4). In 1992, Gabbert and colleagues analyzed the prognostic significance of tumor cell dissociation (TCD) at the invasive front of gastric AC in a cohort of 445 surgically treated stage I–IV patients (20). Even though TCD is not termed TB by the authors, the quantitative assessment of this feature is clearly very closely related to TB as described by Hase and colleagues (46) with TCD grades 0/1 corresponding to absent/mild TB and TCD grade 2/3 to moderate/severe TB. TCD grade 2/3 was found in 51.7% of patients. Interestingly, the degree of TCD at the invasive front predicted aggressive disease with 5-year survival of 38.5% in comparison to 92.3% in patients with absence of this feature, independently of T-stage, tumor size, and grade (*p* = 0.026). Further, patients with high-grade TCD frequently presented with more advanced T-stage, N-stage, vascular invasion, high-grade tumors, and larger tumor size.

**Table 4:** **Studies on tumor budding as a histomorphological prognostic factor in gastric adenocarcinoma**.

Reference	Tumor subtype	Stage	*N*	Treatment	Method for assessment of TuB	Low-grade/high-grade TB%	Correlation of high-grade TB with clinicopathological features (*p* < 0.05)	Outcome
Gabbert et al., 1992 (20)	AC	I–IV	445	445/445 Patients treated by primary resection	Ueno et al., 2004 (22) H&E	48.3/51.7	T-stage, N-stage, tumor grade, circumferential resection margin involvement	OS 31 months in patients with low-grade TB; OS 15 months in patients with high-grade TB (*p* < 0.0001)
Tanaka et al., 2014 (21)	AC	I–IV	320	320/320 Patients treated by primary resection 111/320 Patients received adjuvant CTX 50/320 Patients received palliative CTX	Modified Karamitopoulou et al., 2013 (25) PanCK	40.0/60.0	T-stage, N-stage, L1, synchronous liver metastasis, other distant metastasis, larger tumor size, depressive macroscopic morphology	High-grade TB is a poor prognostic factor in patients with differentiated histology (*n* = 153; HR = 1.61; 95% CI: 1.12–2.41; *p* < 0.01) in univariate analysis. Not independently prognostic in multivariate analysis.

*TB, tumor budding; SCC, squamous cell carcinoma; AC, adenocarcinoma; CTX, chemotherapy; RTX, radiotherapy; H&E, hematoxylin and eosin; PanCK, pancytokeratin; V1, venous invasion; L1, lymphatic invasion; Pn1, perineural invasion; OS, overall survival*.

In a recently published study, Tanaka et al. address TB in a well characterized retrospective cohort of 320 surgically treated stage I–IV gastric AC patients including 161 patients receiving adjuvant or palliative chemotherapy following resection (21). High-grade TB was observed in 60.0% of cases, a similar frequency as reported by Gabbert (20). Importantly, high-grade TB correlated significantly with TNM-relevant features including T-stage, N-stage, lymphatic invasion as well as synchronous liver metastasis and distant metastasis. Further, high-grade TB was associated with larger tumor size and depressive macroscopic morphology. In patients with predominantly differentiated histology (*n* = 153), TB was found to be an adverse prognostic indicator (HR = 1.61; 95% CI: 1.12–2.41; *p* < 0.01) in univariate analysis. However, when including other TNM-relevant features, TB did not retain prognostic significance in multivariate analysis. Taken together, TB may be a possibly highly prognostic feature that is clearly underrepresented in studies of histomorphological features of gastric cancer.

Tanaka and colleagues also provide insight into the biological processes that may contribute to the formation of tumor buds in gastric AC. Using immunohistochemistry, geographic protein expression analysis of the pro-proliferative and pro-invasive tropomyosin-related receptor kinase B (TrkB) in gastric carcinomas was performed. TrkB expression may promote EMT in malignant cells and has been associated with chemotherapy resistance of esophageal carcinomas (61, 62). In consistence with this, Tanaka and colleagues report an increased frequency of TB in patients with high TrkB expression at the tumor center, margin, or invasive front. Tumor buds themselves frequently over-expressed TrkB, confirming previous studies on this protein in tumor buds of colorectal cancer (39). Consequently, over-expression of TrkB expression may be a common feature of cells undergoing EMT and may allow increased resistance to pro-apoptotic stimuli and chemotherapeutic agents.

#### Future outlook

**Figure 3:**
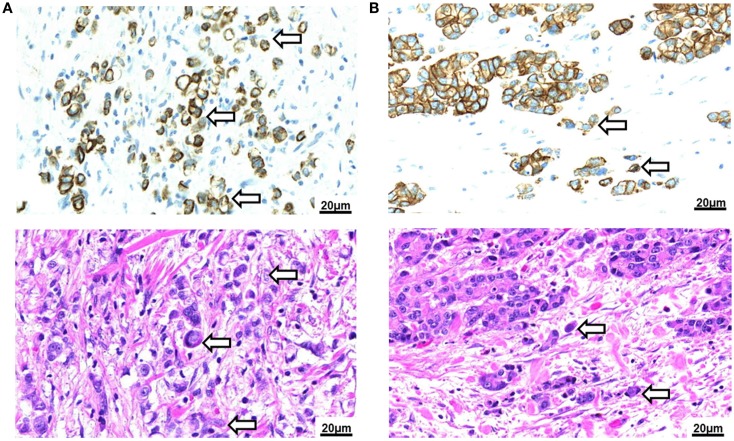
**Tumor budding in Barrett’s carcinoma**. **(A)** Poorly differentiated adenocarcinoma of the esophagus with signet cell component as seen in a pancytokeratin (top) and H&E (bottom) stain (250×). Tumor buds cannot be differentiated by morphology from the diffusely infiltrating tumor mass. **(B)** Intestinal type adenocarcinoma of the esophagus with presence of tumor buds at the invasive front as seen in a pancytokeratin (top) and H&E (bottom) stain (250×).

Data on the prognostic impact of TB in gastric AC is still sparse. More data are needed to reliably judge the potential value of TB for the clinical management and follow-up of gastric cancer patients. Further, the impact of TB in the early-stage subgroup, in pre-operative biopsy specimens and in the adjuvant setting has yet to be addressed. To researchers interested in TB, gastric cancers also pose a challenge due to the higher frequency of poorly differentiated and signet cell carcinomas with a primary dissociative growth pattern. It has so far not been explicitly addressed whether gastric carcinomas or any other cancer of the GI-tract with a primary dissociative growth pattern should be defined as “high-grade TB” (Figure 3A). As signet cell carcinomas of the stomach also frequently display primary E-cadherin mutations, the use of this feature to characterize cells undergoing EMT is limited (63). We believe that differentiation of cancers with a primary dissociative growth pattern from cancers with high-grade TB should therefore be addressed on the base of morphology; the definition of TB as the presence of single cells and small clusters of up to five dedifferentiated tumor cells ahead of the invasive front implicates that a solid tumor body must be present for bona fide TB to occur (64) (Figure 3B). Clearly, the specific characteristics of TB in AC of different histological type and differentiation require further investigation.

## Conclusion

Tumor budding is frequently observed in carcinomas of the upper gastrointestinal tract. It is a valuable tumor-related prognostic factor and indicator of aggressive disease biology. In esophageal and gastric carcinomas, presence of TB in the resection specimen predicts unfavorable clinicopathological features and early disease recurrence. Information on TB provided by the histopathologist could be of particular value for personalized patient management in two distinct clinical scenarios: first, information on TB could be an important factor for risk stratification of patients with locally invasive SCC or AC of the esophagus (15, 19). Consequently, patients with high-grade TB may benefit from esophagectomy rather than treatment with locally ablative procedures. It is fair to assume that assessment of TB in EMR specimens or pre-operative biopsy material could provide important information on the relative risk for lymph node metastasis and disease recurrence. Further studies are therefore of particular importance to address this clinical scenario. Second, TB may be a valuable additional prognostic indicator to aid the selection of high risk patients with esophageal cancer for multimodality therapy. In particular, some patients with pT2 esophageal cancer present with early disease recurrence following resection of the primary tumor. In this setting, TB may provide additional prognostic information for personalized therapeutic approaches. However, a central limitation to these applications for TB in the assessment of esophageal and gastric cancers is still the relative scarcity of data. As compared to colorectal cancer where TB is already officially recognized as additional prognostic factor by the UICC (1), studies on TB in upper gastrointestinal cancers are based on a limited patient number and a small group of study centers. Large multi-centric studies including robust statistical analysis are therefore urgently needed to determine whether TB can assume a similar role in upper gastrointestinal cancers.

Standardization of assessment methods is another central roadblock for the establishment of TB as histomorphological prognostic factor in gastrointestinal pathology. This holds true for carcinomas of the esophagus and stomach and also the colorectum. In analogy to the BRE-score for breast cancer or the Gleason score in prostate cancer, TB shows potential to assume an important role for prognostication and therapy planning in GI-cancers. Consequently, approaches for standardization should not be limited to one section of the GI-tract but the attempt to find consensus should be made on an integrative basis. This requires agreement on both qualitative and quantitative criteria.

For qualitative criteria, broad consent exists on the definition of TB as single tumor cells or small clusters of up to five cells ahead of the invasive front (65). The most widely used approach to visualize this feature is a standard H&E stain, but expert opinion is variable (19, 27, 66). In cases where strong inflammation is present at the invasive front, differentiation of tumor buds from macrophages, cell detritus, ruptured glands, and reactive stromal cells can be challenging. Pancytokeratin immunohistochemistry has been recommended to better identify tumor buds in difficult cases and may allow quantification of TB using digital imaging (66, 67). For quantitative criteria, consensus on a reproducible approach to define low or high-grade TB seems mandatory. A large number of different methods have been suggested for the assessment of TB in daily diagnostic practice, but systematic inter-observer studies are few. To provide evidence on the optimal methodological approach to visualize TB, a systematic comparison of the inter-observer reproducibility in large multi-centric studies is therefore recommended.

## Conflict of Interest Statement

The authors declare that the research was conducted in the absence of any commercial or financial relationships that could be construed as a potential conflict of interest.
